# Leading change in practice: how “longitudinal prebriefing” nurtures and sustains in situ simulation programs

**DOI:** 10.1186/s41077-023-00243-6

**Published:** 2023-01-21

**Authors:** Susan Eller, Jenny Rudolph, Stephanie Barwick, Sarah Janssens, Komal Bajaj

**Affiliations:** 1 Immersive Learning and Learning Spaces, Center for Immersive and Simulation-Based Learning, School of Medicine, Stanford University, 291 Campus Drive, Stanford, CA LK311B USA; 2 Surgery, Health Professions Education, Center for Medical Simulation, Harvard Medical School, Massachusetts General Hospital-Institute for Health Professions, Boston, MA USA; 3 Clinical Education, Mater Education, Mater Misericordiae, Brisbane, Australia; 4 Obstetrics and Gynaecology, Clinical Simulation, Mater Health, Mater Misericordiae, Brisbane, Australia; 5 Obstetrics & Gynecology and Women’s Health, Department of Quality & Safety, NYC H+H Simulation Center, NYC Health + Hospitals/Jacobi, Albert Einstein College of Medicine, Bronx, NY USA

**Keywords:** In situ simulation, Prebriefing, Organizational change, Healthcare quality, Patient safety

## Abstract

In situ simulation (ISS) programs deliver patient safety benefits to healthcare systems, however, face many challenges in both implementation and sustainability. Prebriefing is conducted immediately prior to a simulation activity to enhance engagement with the learning activity, but is not sufficient to embed and sustain an ISS program. Longer-term and broader change leadership is required to engage colleagues, secure time and resources, and sustain an in situ simulation program. No framework currently exists to describe this process for ISS programs. This manuscript presents a framework derived from the analysis of three successful ISS program implementations across different hospital systems. We describe eight change leadership steps adapted from Kotter’s change management theory, used to sustainably implement the ISS programs analyzed. These steps include the following: (1) identifying goals of key stakeholders, (2) engaging a multi-professional team, (3) creating a shared vision, (4) communicating the vision effectively, (5) energizing participants and enabling program participation, (6) identifying and celebrating early success, (7) closing the loop on early program successes, and (8) embedding simulation in organizational culture and operations. We describe this process as a “longitudinal prebrief,” a framework which provides a step-by-step guide to engage colleagues and sustain successful implementation of ISS.

**Supplementary Information:**

The online version contains supplementary material available at 10.1186/s41077-023-00243-6.

## Background

In situ simulation is conducted in the actual care environment [1] and serves as a vehicle of study or a test of change [2, 3]. Simulation practitioners deploy ISS in several ways: system probing for latent threats [4, 5], target training for specific crisis events [6], embedding new system processes [2], assessing safety of new environments [7, 8], and team training [9, 10]. Learning from ISS can occur at the individual, team, unit, or organizational level, with measurable improvements often greatest at the organizational level [11, 12].

In situ simulation (ISS) is the nexus of rival priorities in healthcare systems. The competing demands of clinical performance, efficiency, patient safety, and applied learning often clash when they intersect in frontline ISS. Despite more than 10 years of demonstrated benefits to healthcare systems [13–15], ISS leaders often struggle with how to balance the daily pressures of patient care, staffing levels, and patient throughput with the known benefits of applied practice. To manage this tension, ISS is often framed as a “nice to do,” an add-on, when time permits, in busy clinical units. Understandably, it may be seen as distracting or taking away precious time from overworked clinical professionals.

This produces two challenges for simulation practitioners:

1. When they are not adequately tuned in to the competing priorities, they can be blind sided when their well-intentioned efforts to start and sustain ISS programs are met with reluctance, fear, resentment, or outright refusal [16–18].
2. Health system leaders who have committed space, simulation equipment, staff time and faculty development to improve quality, and safety or staff engagement via these programs may wonder about the return on investment.

Simulation leaders must therefore design for impact across nested organizational levels, in which individuals are nested in groups, nested in departments, nested in organizations which are part of health systems, and influenced by the external environment. A case for simulation that is persuasive at the team or sub-unit level, to be successful, may need to demonstrate impacts at higher levels. And research on change leadership that includes simulation will benefit from a multi-level lens [19].

The simulation literature has focused intently on prebriefing — information sharing for participants that play a role in psychological safety to build engagement for participants, primarily with a focus on during a session that typically immediately follows the prebriefing. Critical actions suggested for prebriefing include the following: clarifying objectives, equipment functionality, roles of the participants and faculty, confidentiality of the session, and expectations of participants during the simulation [11, 12, 14, 20, 21].

Prebriefing ISS teams may pose additional challenges including complexity of scheduling participants within a clinical environment and safety risks related to using real or simulated equipment in clinical areas [17, 18]. While usual guidance on prebriefing may have demonstrated impacts on proximate learning, they often do little to tap into health system “pain points,” or key goals and therefore fail to build either the legitimacy or a compelling narrative that entices busy healthcare workers and leaders to “buy in” to the program. This puts well-designed ISS programs at risk for low enrollment, session cancellations, or even defunding — outcomes many simulation leaders have learned the hard way. Standard recommendations for prebriefings for incenter or ISS temporally proximate to the learning session are necessary, but not sufficient to guide the long-term factors that influence organizational adoption ISS.

This paper therefore explores the question, “How did three disparate ISS programs address the challenge of linking in situ simulation to the concerns and goals of health system colleagues?” We present a retrospective description of our evolving practice of managing and leading ISS programs and apply Kotter’s theory of change leadership [22] to categorize and illuminate the processes we noticed empirically. These efforts yielded a change leadership framework [23] we call “longitudinal prebriefing.”

## Methods: learning from each other and practice

The author group convened based on dialogue prompted by postings on an online journal club about in situ simulation (Simulcast, September 2019). Authors S. E., S. B., S. J., and K. B. described establishing programs at three locations, encountering comparable institutional and learner barriers, and using similar strategies to aid implementation. These anecdotal comparisons of successes inspired a more formal exploration of the processes to see if we could identify a framework to guide others in ISS implementation. We inductively analyzed emergent practices that authors S. E., S. B., S. J., and K. B. developed and adapted as they rolled out ISS programs in three settings in North America and Australia. Program A, in a large health system in Queensland, Australia, employed ISS to evaluate and improve code team performance at an urban academic medical center on the east coast of the USA, utilized ISS to improve performance on obstetrical emergencies; program B, at an urban academic medical center on the east coast of the USA, utilized ISS to improve performance on obstetrical emergencies; and program C used ISS to improve adherence to national resuscitation guidelines in a neonatal intensive care unit at a Midwestern United States Medical Center. Analyzing programs from three unique contexts allowed the author team to consider knowledge gained from their own programs as insiders and also view the other programs as outsiders in alignment with comparative qualitative methodology [24]. Reporting on ethical considerations pertaining to human subjects is an important consideration for disseminating academic knowledge; due to the de-identified nature of the narratives, we did not seek IRB approval for this work when we initiated our analysis.

### Change leadership as insiders

Most research reported in biomedical and health professions education literature is “outsider” research conducted from an observer perspective [25]. In this dominant paradigm, the legitimacy of findings relies on the “objectivity” of the researcher standing outside of the phenomena under study [26, 27]. However, participatory action research traditions have nurtured an alternative known as “insider” research [28]. This is research by “actors immersed in local situations generating contextually embedded knowledge that emerges from experience” [29] (p. 60). This allows for “pre-understanding” of context, relationships, and organizational processes. In our analysis, our team’s insider status was beneficial in several ways. Firstly, our situated understanding enabled us to identify individual, unit, and organizational pain points to customize program priorities. Secondly, our insider status allowed us to analyze relationships and internal social networks to build a coalition to support the program and enroll participants. Thirdly, we were able to build on knowledge of other people’s roles and scope of practice to analyze how these interacted and influenced program implementation at multiple levels. Following recommendations from the insider research literature [24], we analyzed our own ISS implementation journeys combining both insider experiential and outsider theoretical knowledge to reframe and generalize our understanding of the processes deployed in ISS programs we developed.

### Identification of common themes

To better compare our ISS implementation narratives, each site wrote their respective approach without any specified form/structure other than removing all identifiable information. In reconstructing timelines and developing their narratives, the authors reviewed documentation from their own institutions, such as emails, meeting minutes, instructor notes, flyers for participants, and reports of successes shared within our institutions or at professional organizations/conferences. All narratives and subsequent analyses were written on Microsoft Word documents and shared between the authors in a secure online portal. These narratives were then reviewed as a group and analyzed initially using thematic analysis [30, 31] combined with comparative analysis strategies [24].

Data analysis followed the six-step thematic analysis process as outlined by Braun and Clarke [30]. Authors SE, SB, SJ, and KB first familiarized themselves with all of the narratives and writing memos to distill and sharpen key prebriefing processes [32]. In the second phase, we selected key phrases to generate the initial codes for actions [30]. The group next searched these codes for similarities to determine if there was any natural clustering that represented themes [30]. During this third phase, the authors found that similar activities had been labeled differently depending upon the context. This discussion led to adopting insider comparative qualitative analysis methodology of alternating between analytical closeness and analytical distance [24]. This strategy allows for those with in-depth knowledge to compare and contrast data between contexts in order to develop a collective understanding of the processes [24] and shared terminology. In the fourth thematic analysis phase of reviewing themes [30], the authors mapped themes/steps on an ISS implementation timeline. Comparing timelines across the various contexts revealed that although the actual length of time between steps varied, the order remained consistent. This shared understanding guided the next phase of defining and naming the themes [30] so they were relevant across contexts. These themes described a process for implementing ISS within an organization: identifying motivation for program development; obtaining buy-in from organizational leaders and participants; establishing program goals, branding, and promoting the program; educating the organization on simulation/ISS; planning for successes; closing the loop on any issues found; and formally embedding ISS program into the organization. Reviewing these inductive, empirically generated themes, the group realized they paralleled Kotter’s prescriptions for organizational change leadership in many ways.

These themes were then compared against Kotter’s change leadership steps to adapt prescriptions for the simulation community based on the authors’ collective experiences. The final phase of thematic analysis is producing the report [30], and the longitudinal prebriefing process we outline here describes our adaptation of Kotter’s theory of change leadership to categorize and illuminate the processes we noticed empirically as we built our programs. Digital supplement 1 provides snapshots of the program narratives as well as examples of how each program executed the longitudinal prebrief. Digital supplement 2 provides additional details regarding the phases of thematic analysis.

### Trustworthiness

Several techniques were employed to enhance the trustworthiness of our data analysis [33]. During thematic analysis, the initial author group maintained an audit trail in their secure online portal. The authors used documentation from their ISS implementations to reconstruct their narratives to minimize hindsight bias. Author JR was invited to review the methodology, thematic codes, and the table for longitudinal prebriefing. By cross-walking the final themes back to the raw data, author J. R.’s review provided additional verification of the themes and actions described in the process table. Our author group reviewed the initial narratives after the manuscript, table, and figure were developed to ensure adequate description of the process and prevent confirmation bias by leaving out or misconstruing concepts that cross-walked Kotter’s model to the processes we describe.

We would like to note there might be some limitations to our conclusions drawn via these methods because of our insider status and commitment to change leadership. Our research team was highly motivated to identify themes and patterns to clarify the ISS work we had done. While this served as an engine for the work, it subjected us to “motive-driven cognition,” i.e., we were at risk for arriving “at a particular conclusion, attempt to be rational, and to construct a justification of [our] desired conclusion that would persuade a dispassionate observer” [34] (p.272). To mitigate this sort of bias, we worked in good faith and with significant rigor to analyze each other’s program data (not just our own) and to have a member of the team not involved in any of the programs reanalyze in the thematic analysis.

## Applying Kotter’s model of change leadership to ISS

**Fig. 1:**
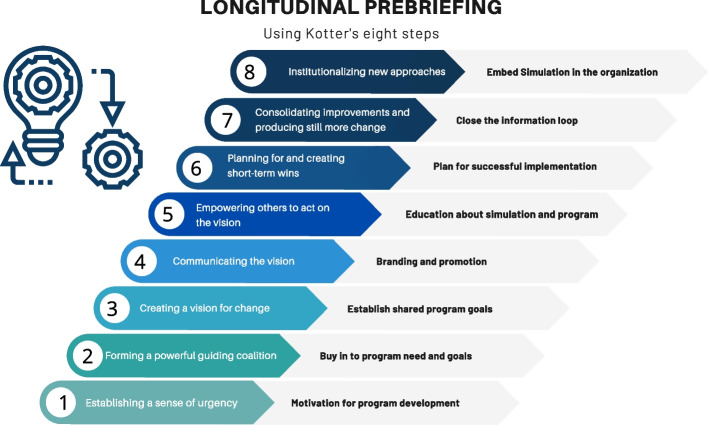
Longitudinal prebriefing

In his seminal work “Why Transformation Efforts Fail” [22], Kotter describes eight steps to organizational transformation based on his study of over 100 companies of varying sizes and achievement. As each author shared their institution’s ISS implementation journey, it was clear that significant engagement and culture change, the hallmarks of organization transformation, were required for success. Longitudinal prebriefing, which begins well before initiation of any simulations, helped to accelerate the necessary engagement and culture change. Figure 1 summarizes our theory elaboration of Kotter’s eight transformational steps applied to ISS. Below we describe the Kotter’s eight steps in detail and describe the elements highlighted in the thematic analysis of our ISS programs that relate to each step. This is also summarized in Table 1 along with empirical examples from the program narratives.

**Table 1:** Adaption of Kotter’s change leadership for longitudinal prebriefing

Leadership step	Change leadership for ISS	Illustrations
**Establish a sense of urgency**	Identify “pain points” or precious goals of key stakeholders to inspire action and program development	○ Leverage patient stories, moral imperatives, regulatory requirements, or institutional threats ○ Multiple incident reports of adverse outcomes for babies due to communication issues identified in neonatal resuscitations ○ National accreditation requirements identified for training in recognition and response to deteriorating patients
**Form a guiding coalition**	Engage top-down and bottom-up multi-professional partners to create buy-in. Do not work alone	○ Simulation facilitators as well as clinical stakeholders and healthcare leaders form a working party to clarify goals of ISS program (e.g., clinical readiness, quality, inclusion) ○ Gain representation on relevant hospital committees to garner support across different levels of governance
**Create a vision**	Co-create shared goals and vision to address individual, unit, and organisational needs	○ Host regular meetings with program sponsors/supporters to gather input into program scope and establish a shared mental model on program goals and expectations ○ Utilize evidence-based examples in the literature to inform goals
**Communicate the vision**	Communicate shared goals, vision, and outcomes that accurately represent value and assist with positive reinforcement of participation	○ Brand program with a “catchy name” that communicates the function and vision of the program ○ Socialize program in a variety of settings, including existing forums such as grand rounds or safety huddles
**Empower others to act on the vision**	Energize (or re-energize) program participants by clarifying program logistics and providing approachable opportunities for familiarization with the program	○ Commence program with “fun” simulation-based activities within the clinical environments to introduce departments to the program and educate them about simulation ○ Provide early adopters who want to learn more about simulation/debriefing professional simulation development opportunities
**Plan for and create short-term wins**	Identify and celebrate early adopters and early successes to build momentum	○ Commence program in clinical units that are excited, engaged, and who have had previous positive experiences with ISS ○ Publicly advertise the date, time, and the scenario theme, in advance, for the initial program commencement period
**Consolidate improvements to produce still more change**	Ensure program impacts are visible by closing the loop with multi-professional partners	○ Communicate identified issues uncovered during the simulations with hospital leadership ○ Share improvements at staff huddles ○ Create infographics to communicate improvements
**Institutionalize new approaches**	Embed simulation in the organisational culture and regular operations	○ Include ISS programs in specific policy documents on workforce training/development ○ Establish formal reporting process/agenda item at quality and safety or hospital governance meetings ○ Celebrate program successes through annual anniversaries of program commencement and dissemination of annual reports

*Establish a sense of urgency* in ISS implementation mirrors Kotter’s first step since successful implementation of ISS programs requires impetus to overcome organizational complacency. Simulation change agents often find that “no urgency-no buy-in.” Potent drivers of change can include “pain points” such as poor performance on benchmarked quality indicators, financial impacts, trends from morbidity and mortality reports, and poignant stories of patient outcomes [22, 35]. Exploring our institutional stories identified these factors as successful drivers for ISS buy-in: unfavorable outcomes in maternal hemorrhage, gaps in training for emergency response teams, and unfamiliarity with equipment and national resuscitation standards. The nature of the problems addressed then drives selection of ISS program guiding coalition of stakeholders.

Kotter’s second step, *Form a powerful guiding coalition*, emphasizes that sustainable change requires a strong, thoughtfully composed team [22]. It is essential to enlist a diverse team of bottom-up and top-down multi-professional organizational champions [36, 37]. This coalition provides formal and informal leadership, credibility, clinical expertise, and power to remove barriers [22]. An organization’s visible commitment to improving culture and supporting simulation activities can improve the psychological safety and engagement of participants [16]. Our analysis yielded examples as follows: gaining support from nursing unit managers and educators, including members of hospital resuscitation committee, and recruiting local sim-friendly participants as early adopters of the ISS program. Coalition members co-create overall ISS program goals.

After program goals and scenario objectives are established, the third step is *Create a vision* [22]. This step establishes purpose and direction, motivates people to engage, and coordinates actions of team members; when healthcare team members perceive new programs as relevant to their daily work, they are more amenable to changing for improvements [38]. All authors identified a critical component as clarification of the program purpose, explicating what the ISS program was and was not intended to achieve. Interprofessional participants at all institutions expressed anxiety regarding participation, and declaring the lack of formal evaluation during ISS alleviated simulation reluctance. A shared mental model of ISS program goals and expectations facilitates psychological safety, mitigates the risks of simulation in the actual clinical environment [39], and expedites program progression.

In *Communicate the vision*, Kotter advocates for key elements to conveying the message, including simplicity, repetition, and use of multiple forums [22]. One aspect of longitudinal prebriefing was developing titles/branding that communicated the function and vision of the programs, and each of the program described pithy titles during our comparative analysis. All three institutions reported presenting at various levels: grand rounds, leadership meetings, nursing orientation sessions, and unit-based educator forums. Another common feature to our communication strategies was visits to the local unit. After these initial information sessions, we all progressed to further simulation activities with potential participants.

*Empower others to act on the vision* often involves removing structural barriers and providing training as needed to accomplish change [22]. Our shared experiences found the need to remove cultural barriers that included concerns about scheduling or resources and reluctance to participate in ISS. Including the unit leaders in the coalition ameliorates some of the scheduling and supplies, by having mutually agreed-upon go or no-go parameters for running ISS on the unit. Creating fun simulation-based activities for unit participants overcame resistance to participation. Examples of such activities included pop-up simulations with nonthreatening CPR games, scavenger hunts, and Olympic-style games for infrequently used equipment. Providing familiarization to the equipment and ground rules of simulation prior to ISS decreased both anxiety and prebrief time on the day of the event. Another empowerment activity that enhanced psychological safety was offering debriefing courses to educators or early adopters, to increase their comfort with this vital component of ISS.

*Plan for and create short-term wins* is another essential step for successful ISS program implementation. Delivering evidence that culture change efforts yield positive results rewards early adopters, encourages further participation, and motivates managers to support program continuation [22]. Part of planning for short-term wins is identifying early adopters and targeting ISS implementation to those units first. Detecting system issues that could lead to safety errors promoted instant changes that improved care for all patients and not just the simulated event. Another significant short-term win was reporting back to participants on changes to unit practice, or the ISS program, based on their feedback.

The next adapted step in longitudinal prebriefing is *Consolidate improvements to produce still more change*. During this phase, the coalition uses momentum from short-term wins to address more challenging organizational issues [22]. Gains from improving CPR quality of rate and depth led to deeper exploration of leadership issues during resuscitation and reluctance to speak against hierarchy when performance/practice gaps occur. Feedback on successes from ISS should be communicated at multiple levels of the organization. Our strategies included sharing at unit and educator meetings, setting up whiteboards to display successes to entire organization, and celebrating time and participation milestones.

The final step *Institutionalize new approaches* [22] involves having ISS formally embedded in organizational policy. Providing reports of safety improvements allowed formal acknowledgement of success and rationale for institutional change. Our groups reported having ISS as a formal standing agenda item at resuscitation committee meetings, obtaining organizational funding for continuing the ISS program, and adoption of ISS as mechanism for addressing other organizational quality issues. Investing time in longitudinal prebriefing for ISS yielded successful simulation-based programs, quality improvements, and organizational changes.

### Impact of insider status: benefits and limitations

Our author group’s insider status impacted the understanding of context, organizational processes, and relationships to develop the Kotter simulation-specific adaptations presented. As an example of the importance of insider insights into context, the initiation of program A was catalyzed by the need to hardwire protocols to address obstetric emergencies, identified by a series of unfavorable clinical events. This “insider” perspective on the impetus behind program A’s development allowed for a nuanced understanding of the context under which external and internal organizational priorities created urgency, details that might otherwise be missed by an outsider trying to gain understanding of those priorities (and how they evolved) retrospectively.

Program B is an example of how insiders’ understanding of organizational processes may provide an advantage over traditional “outsider” research. The authors involved with this program had deep familiarity with the organization and its processes of cardiac arrest response, since they practiced within the organization. This afforded them a practical understanding of the various code team compositions and the clinical governance structures surrounding code team responses. This familiarity ensured simulations could be tailored to specific unit and code team requirements and facilitated rapid authorization of process improvements suggested following simulation activities.

While reflecting on the implementation of the cardiac arrest ISS program, this “insider” perspective on organizational processes allowed clear elucidation of what processes were no longer adequate, where “business-as-usual” needed change, and the steps required to hardwire that change.

As “insiders,” the authors also have a unique understanding of both healthcare team’s structures and relationships. Preexisting relationships that simulationists had with nursing educators, residency coordinators, and hospital leadership accelerated the implementation of the program C. Nursing and physician educators expressed discomfort with conducting the simulations and agreed to take a simulation instructor course based on positive relationships with the simulation leads. These courses increased the NICU educators’ familiarity with simulation and built trust to within the ISS coalition. Based on negative previous experiences or unfamiliarity with the equipment, some nurses and pediatric residents working in the NICU expressed reluctance to participate in the simulations. The simulation leads leveraged the trust with the NICU nurse and residency educators to offer mini briefing sessions to familiarize participants with the equipment and define expectations. This positive exposure to simulation decreased the reluctance to participate and built trust with the ISS program leads.

The conclusions and recommendations presented here are based on both analyzing our own initiatives as insiders (S. B., S. J., K. B., S. E.) and analyzing each others’ initiatives as outsiders (all the authors). We appreciate that there are threats to the trustworthiness of our conclusions. As seen through the lens of outsider research, S. B., S. J., S. E., and K. B.’s insider status, and particularly their personal investment in the success of their programs, and lack of blinding to outcomes introduce crucial biases. While these threats to our interpretations of our practice-based data exist, rather than seeing them as defects, we argue that they are assets. The inside researchers’ investment in change and richly contextualized understanding where it succeeded and failed complements outsider research by highlighting the lived experience and dilemmas of taking action in real-life contexts.

### Application to other projects

Historically, simulation centers and programs were built on the old adage of “build it and they will come,” but such advice can be detrimental for ISS programs. Our insider research clearly highlights that “how you build it” matters, and time should be spent “playing the long game” in considering change leadership principles for successful ISS implementation. The longitudinal prebrief provides a road map for successful ISS organizational integration. The generalizability of the change leadership framework we propose here is limited by location of the initiatives: North America and Australia. It would be useful to explore how and if change leadership in simulation would be different in more hierarchical or collectivist cultures [40].

Beyond application to the three programs described, the authors have since utilized “The Longitudinal Prebrief” to implement other ISS programs in different contexts and locations. All authors have provided support for other teams initiating ISS programs and activities across geographically dispersed health facility locations by providing guidelines and mentorship that included Kotter’s leadership steps adapted for ISS. Authors S. B. and S. J. utilized this model to coach an ISS program implementation in a smaller regional facility. A key factor to this process was gaining organizational motivation by first spending time understanding the specific clinical and workforce need and connecting the ISS program as a solution to the identified needs. Similarly, author J. R. utilizes the guiding principle “no urgency-no program” when consulting with groups that wish to utilize ISS. Author S. E. developed a policy of ensuring all clinical educators involved in ISS were part of the guiding coalition by having them assist with program design and complete simulation instructor training. Author K. B. strengthened institutional buy-in by co-developing “no-go considerations” for ISS activities that empowered unit leaders and published the framework to provide guidance for others [39]. Components of “longitudinal prebriefing” have also been the subject of faculty development programs at various international conferences, which have been of great interest to the simulation community of practice.

While not specifically naming Kotter’s change leadership principles, many ISS program descriptions report elements of the process we describe in successful implementation of programs for interprofessional health teams. Wheeler et al. described the successful implementation of an ISS program for the deteriorating patient in a pediatric hospital [10]. Change leadership steps described include creating and sharing a clear vision for the program, the presence of a guiding coalition, planning for early gains, and leveraging early success to consolidate change [10]. Riley et al. described using real adverse outcome data to establish a sense of importance for buy-in for an ISS program, as well as feeding back improvements to maintain momentum [41]. Kumar et al. have noted the importance of change management in sustaining simulation programs, highlighting the importance of institutional “buy-in” [42]. These examples highlight how successful ISS programs have instinctively utilized elements of change leadership to embed programs and ensure sustainability. However, to our knowledge, thus far, there has been no comprehensively described approach to ISS program implementation.

## Conclusion

Failing to “play the long game” in simulation program initiation is the norm, rather than the exception. Focusing on short-term gains, putting out fires, and focusing on the urgent at the expense of the important set simulation program leaders up frustration and even burnout. We therefore have differentiated “prebriefing” that is designed primarily as a process temporally proximate to an upcoming simulation session and designed to establish an engaging learning environment for current learners, from a longitudinal briefing process that focuses on building program legitimacy via connections with the politics and priorities of the larger organization. To succeed in the long game, a more comprehensive approach is required to engage colleagues at all levels and ensure that organizations can implement and sustain simulation programs [43]. This longitudinal prebrief focuses on both unleashing colleague’s intrinsic motivation [44] and building the political and clinical credibility of the program. It provides a road map both for linking with the priorities of the larger organization as well as establishing a safe and engaging learning environment for participants.

We modified a well-known organizational change leadership framework to clarify the specifics of organizational engagement needed for successful implementation of ISS. We cataloged and thematically analyzed our program descriptions to adapt Kotter’s framework and provided recommendations for each step. While our approach is limited by the benefits and biases of describing our own experiences, we believe the framework could provide a structured approach for others implementing ISS programs. To test the soundness of this approach in other contexts, we hope that additional examples of activities relevant to each step (and their relevant success or failure) might be reported by other authors to build collective knowledge for best practice ISS implementation. We encourage those who are developing new ISS programs or expanding current programs to experiment with a “longitudinal prebriefing” to their program planning and implementation.

## Supplementary Information

**Additional file 1. Supplement 1.** Descriptions of 3 different in situ simulation programs and examples of longitudinal prebriefing related to each change leadership step.

**Additional file 1. Supplement 2.** Phases of thematic analysis.

## Data Availability

The datasets used and/or analyzed during the current study are available from the corresponding author on reasonable request.

## Abbreviation

ISS: In situ simulation
