# Supplementary material for: Leading change in practice: how “longitudinal prebriefing” nurtures and sustains in situ simulation programs
Source: Adv Simul (Lond). 2023 Jan 21;8:3. doi: 10.1186/s41077-023-00243-6 (PMC9862849; doi:10.1186/s41077-023-00243-6)
Supplement: Supplementary file 1 — Additional file 1. Supplement 1. Descriptions of 3 different in situ simulation programs and examples of longitudinal prebriefing related to each change leadership step. [file 41077_2023_243_MOESM1_ESM.docx]

**Supplement 1: Descriptions of 3 different in situ simulation programs and examples of longitudinal prebriefing related to each change leadership step**

**Program A:**

An identified need to train the hospital's emergency response teams resulted in the development and implementation of an organisational wide in situ simulation program. The planning group knew that historically ‘mock codes’ were not routinely conducted and were not received positively. A simulation team, who were trained in simulation delivery, led the development of this program and an interprofessional group came together to discuss program goals and plan implementation. The group had representation on the hospital resuscitation committee who provided formal support of the program. A grant from the hospital foundation was secured, which provided financial for the program set up costs. The lead of the in situ simulation program gathered input to the program goals, implementation, and scenarios, and gained support from the nurse unit managers, educators, and local staff. The program was branded with a ‘catchy name’ that communicated the function and vision of the program. The simulation team also regularly delivered presentations about the program, its goals and its outcomes at leadership meetings. For two months prior to commencing the in situ simulation program, the team conducted ‘fun’ CPR challenges in the clinical setting to introduce departments to the program and education them about simulation. Clinical departments that were known supporters/adopters hosted the in situ simulations during the initial program implementation. For the first two months of the program, staff were notified of the simulations in advance including date, time, and scenario. The in situ simulations were aimed to be set up, delivered, and debriefed in 30-40 minutes to enable participation of busy clinical staff. Post the simulation activities a report was compiled and shared with key stakeholders and was a standing agenda item at the resuscitation committee meeting. Attendance at this program was supported through inclusion in specific policy documents. After the 1000th participant, there was celebration that included social media, internal news stories and individual thank you cards for participants.

**Program B**

Unfavourable outcomes related to maternal haemorrhage had demonstrated a need to establish and implement an organisational wide in situ simulation program. The system had already seen uptake and success with center-based activities and the simulation expertise was ready to extend into the clinical areas. Several months before conducting the first in situ simulation, a multi-disciplinary interprofessional core team was assembled, anyone who volunteered was automatically included. The objectives of the program were clarified and a program scope was sharpened. The core team met with sponsors to gather input into program scope and have a shared mental model on program expectations. A three-part joint Grand Rounds series was developed and executed which included 1) Simulation/Debriefing related discussion regarding what the in situ program was (and wasn’t), what to expect and orientation to the equipment 2) fundamentals of maternal haemorrhage and a review of local Policy &Procedure, which in itself identified a need for a policy and procedure update. Written communication (email and flyers) reinforcing intention of the program also occurred. Prior to initiating haemorrhage-related simulations, the team did a some “fun” skill-building activities – a scavenger hunt, a “set-up the rapid infuser” Olympics. The schedule for the first 20 simulations was publicly posted well in advance. Early adopters who wanted to learn more about simulation/debriefing were offered the opportunity for professional development, including a 1-day debriefing course. A large whiteboard in a staff breezeway was installed and regular updates regarding the program were included. On the one-year anniversary of the program, we celebrated by disseminating an annual report which included photos and had cake (that said “Happy Birthday Simulation Program!”). An annual grand rounds program morphed into a bi-annual simulation fairs. As the program shifted to address other emergencies, the above longitudinal prebrief was completed again.

**Program C**

Recurrent communication challenges during neonatal resuscitations resulting in issues with adherence to the neonatal resuscitation protocols (NRP) highlighted the need for a focussed in situ simulation program. The residency program director and staff nurse educator approached the simulation center team to perform monthly training sessions on high-risk events. Intended participants for the scenarios included: neonatal intensive care fellows, paediatric residents, staff nurses, pharmacy, and respiratory therapists. The program coordinators initially encouraged early adopters, who had previous positive experiences with in situ simulation to volunteer to participate. To gain buy in from reluctant staff, the simulation center leaders met with the neonatal intensive care unit (NICU) leaders to clarify objectives and differentiate simulation for learning vs sim for assessment (which this was NOT) for the exercise and agree upon confidentiality for the learners. The nurse staff educators and residency directors were invited to take a simulation instructor course to learn scenario design and debriefing techniques. The simulations were introduced to all new NICU nurses during nurse orientation sessions. During these simulation sessions, expectations for roles/participation, and confidentiality of simulations was discussed. Previous, negative experiences of simulation was also debriefed and a reset of how simulation would run in the NICU was achieved. The Simulation Center went to the NICU once a month when paediatric residents first rotated back to the unit to conduct brief simulation sessions focussing on bag-valve masking, intubation, and CPR. Information sheets were posted in the break room and sent electronically to staff, outlining the timeline (monthly dates) and goals of the simulations. The in situ simulations uncovered several system and communication errors; these issues were communicated with the NICU leadership and addressed. These successes were shared during the monthly pre-simulation huddles and posted on the unit for all to celebrate.


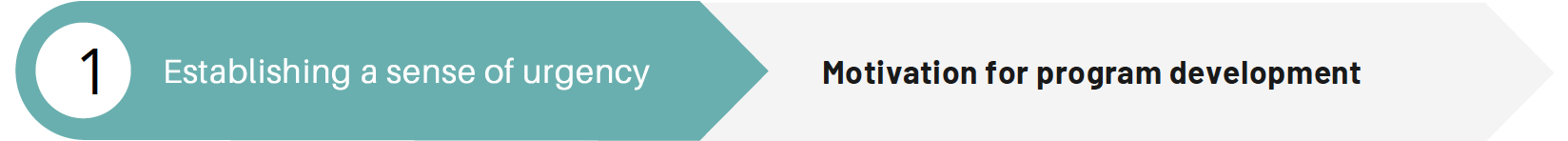


| Program | Longitudinal Prebriefing Action |
| --- | --- |
| A | An identified need to train the hospital’s emergency response teams to support a national standard and patient management resulted in the development and implementation of an organisational wide in situ simulation program. |
| B | Unfavourable outcomes related to maternal haemorrhage had demonstrated a need to establish and implement an organisational wide in situ simulation program. |
| C | Recurrent communication challenges during neonatal resuscitations resulting in issues with adherence to the NRP protocols highlighted the need for a focussed In situ simulation program. |


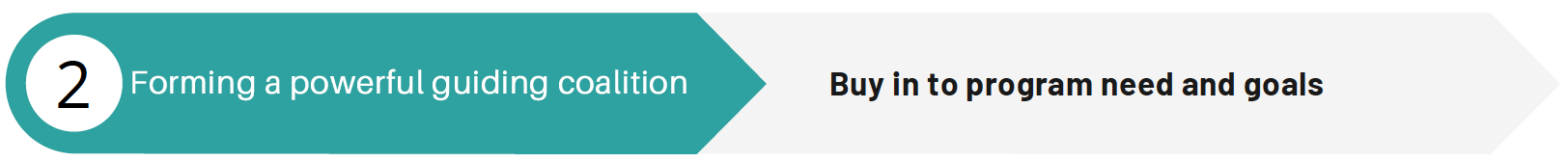


| Program | Longitudinal Prebriefing Action |
| --- | --- |
| A | The simulation team led the development of this program and an interprofessional group came together to discuss program goals and plan implementation. The group had representation on the hospital resuscitation committee who provided formal support of the program. The lead of the in situ simulation program gathered input to the program goals, implementation, and scenarios, and gained support from the nurse unit managers, educators and local staff. |
| B | Several months before conducting the first in situ simulation, a multi-disciplinary interprofessional core team was assembled, anyone who volunteered was automatically included. The objectives of the program were clarified, and a program scope was sharpened. |
| C | The residency program director and staff nurse educator approached the simulation center team to perform monthly training sessions on high-risk events. The nurse staff educators and residency directors were invited to take a simulation instructor course to learn scenario design and debriefing techniques. |


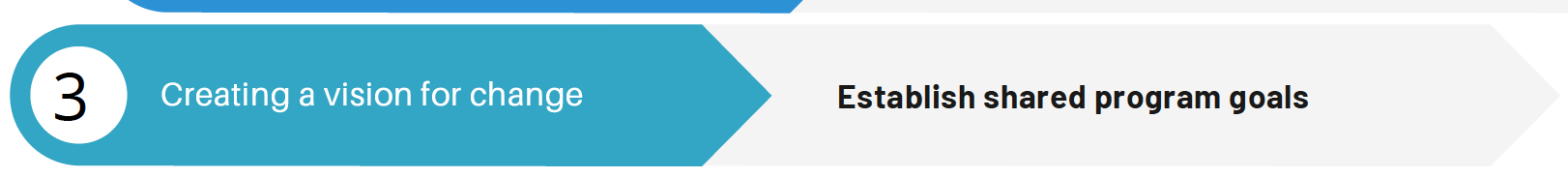


| Program | Longitudinal Prebriefing Action |
| --- | --- |
| A | The lead of the in situ simulation program gathered input to the program goals and scenarios, and gained support from the nurse unit managers, educators and local staff. |
| B | Meeting with sponsors to gather input into program scope and have a shared mental model on program expectations. |
| C | To gain buy in from reluctant staff, the Simulation Center leaders met with the NICU unit leaders to clarify objectives and differentiate sim for learning vs sim for assessment (which this was NOT) for the exercise and agree upon confidentiality for the learners. The nurse staff educators and residency directors were invited to take a simulation instructor course to learn scenario design and debriefing techniques. |


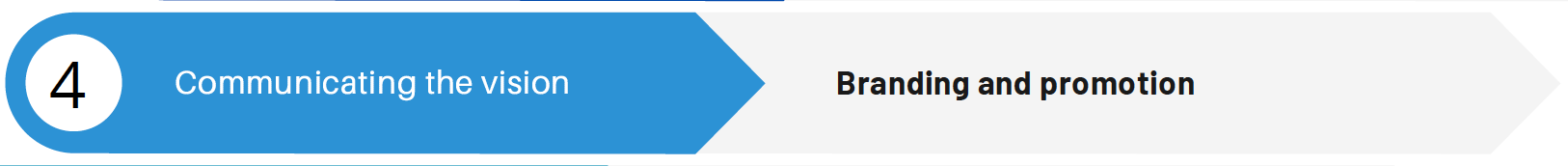


| Program | Longitudinal Prebriefing Action |
| --- | --- |
| A | The program was branded with a ‘catchy name’ that communicated the function and vision of the program. The simulation team also regularly delivered presentations about the program, its goals and its outcomes at leadership meetings. For two months prior to commencing the in situ simulation program, the team conducted ‘fun’ CPR challenges in the clinical setting to introduce departments to the program and education them about simulation. After the 1000th participant, there was celebration that included social media, internal news stories and individual thank you cards for participants. |
| B | A three-part joint Grand Rounds series was developed and executed which included 1) Simulation/Debriefing related discussion regarding what the in situ program was (and wasn’t), what to expect and orientation to the equipment 2) fundamentals of maternal hemorrhage and a review of local policy & procedure, which in itself identified a need for a policy and procedure update. Written communication (email and flyers) reinforcing intention of the program also occurred. |
| C | •Information sheets were posted in the break room and sent electronically to staff, outlining the timeline (monthly dates) and goals of the simulations. |


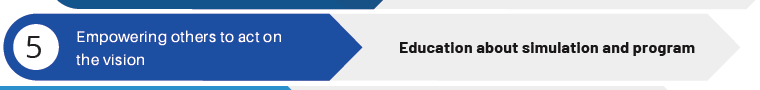


| Setting | Longitudinal Prebriefing Action |
| --- | --- |
| A | For two months prior to commencing the in situ simulation program, the team conducted ‘fun’ CPR challenges in the clinical setting to introduce departments to the program and education them about simulation. For the first two months of the program, staff were notified of the simulations in advance including date, time, and scenario. The in situ simulations were aimed to be set up, delivered and debriefed in 30-40minutes to enable participation of busy clinical staff. |
| B | Early adopters who wanted to learn more about simulation/debriefing were offered the opportunity for professional development, including a 1-day debriefing course. |
| C | The nurse staff educators and residency directors were invited to take a simulation instructor course to learn scenario design and debriefing techniques. The Sim Center introduced all new NICU nurses to New-B during nurse orientation sessions. During these simulation sessions, expectations for roles/participation, and confidentiality of simulations was discussed. Previous, negative experiences of simulation was also debriefed and a reset of how simulation would run in the NICU was achieved. The Sim Center went to the NICU once a month when pediatric residents first rotated back to the unit to conduct brief simulation sessions focussing on bag-valve masking, intubation, and CPR. The newly hired nurses, respiratory therapists, and pharmacists joined in these sessions and NEW-B was used to continue familiarisation with the mannequin. |


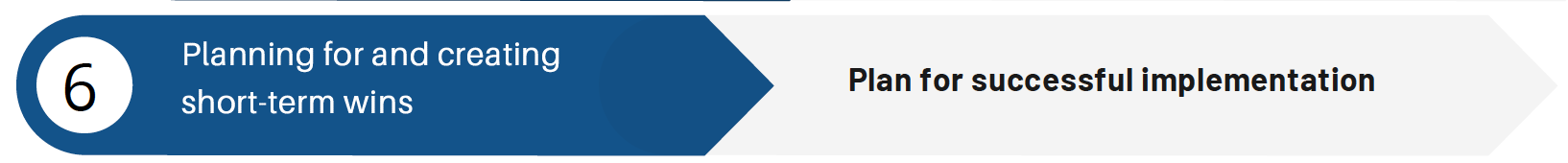


| Setting | Longitudinal Prebriefing Action |
| --- | --- |
| A | Clinical departments that were known supporters/adopters hosted the in situ simulations during the initial program implementation. For the first two months of the program, staff were notified of the simulations in advance including date, time, and scenario. The in situ simulations were aimed to be set up, delivered, and debriefed in 30-40minutes to enable participation of busy clinical staff. |
| B | Prior to initiating haemorrhage-related simulations, the team did a some “fun” skill-building activities – a scavenger hunt, a “set-up the rapid infuser” Olympics. The schedule for the first 20 simulations was publicly posted well in advance. Early adopters who wanted to learn more about simulation/debriefing were offered the opportunity for professional development, including a 1-day debriefing course. |
| C | The program coordinators initially encouraged early adopters, who had previous positive experiences with in situ simulation to volunteer to participate. Information sheets were posted in the break room and sent electronically to staff, outlining the timeline (monthly dates) and goals of the simulations. |


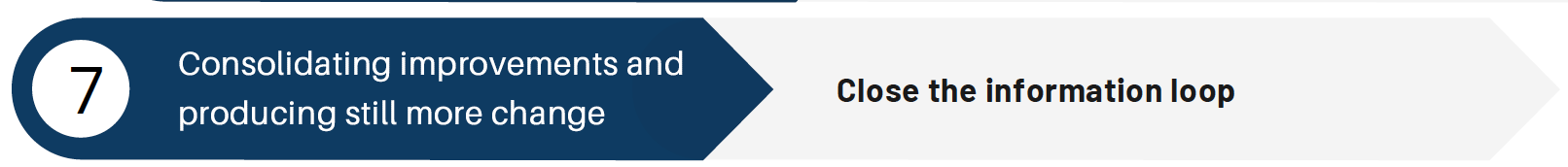


| Setting | Example |
| --- | --- |
| A | Post the simulation activities a report was compiled and shared with key stakeholders and was a standing agenda item at the resuscitation committee meeting. |
| B | A large whiteboard in a staff breezeway was installed and regular updates regarding the program were included |
| C | The in situ simulations uncovered several system and communication errors; these issues were communicated with the NICU leadership and addressed. These successes were shared during the monthly pre-simulation huddles and posted on the unit for all to celebrate. |


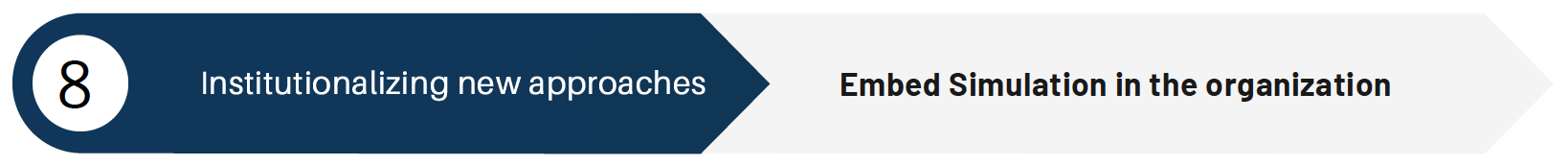


| Setting | Example |
| --- | --- |
| A | Post the simulation activities a report was compiled and shared with key stakeholders and was a standing agenda item at the resuscitation committee meeting. Attendance at this program was supported through inclusion in specific policy documents. |
| B | On the one-year anniversary of the program, we celebrated by disseminating an annual report which included photos and had cake (that said “Happy Birthday Simulation Program!”). An annual grand rounds program morphed into a simulation fair. |
| C | The in situ simulations uncovered several system and communication errors; these issues were communicated with the NICU leadership and addressed. These successes were shared during the monthly pre-simulation huddles and posted on the unit for all to celebrate. |
