# Supplementary material for: Leading change in practice: how “longitudinal prebriefing” nurtures and sustains in situ simulation programs
Source: Adv Simul (Lond). 2023 Jan 21;8:3. doi: 10.1186/s41077-023-00243-6 (PMC9862849; doi:10.1186/s41077-023-00243-6)
Supplement: Supplementary file 2 — Additional file 1. Supplement 2. Phases of thematic analysis. [file 41077_2023_243_MOESM2_ESM.docx]

| Phases of Thematic Analysis (1) | Comparative qualitative descriptions(2) of process for longitudinal prebriefing |
| --- | --- |
| Phase 1: Familiarizing yourself with your data | Wrote narratives for each ISS program and spending time reading each other’s independently. Highlighted topics of interest |

| Phase 2: Generating initial codes | Met online and sharing our initial thoughts of highlighted areas – developed initial codes. |
| --- | --- |

.

| Phase 3: Searching for themes | Looked for clusters that suggested themes across narratives. During this discussion we found similarities in the programs although they had been called different things in each setting. Some of the initial codes were then modified and themes identified and named. |
| --- | --- |

| Phase 4: Reviewing themes | Plotted themes along longitudinal timeline as to when in each program these activities were carried out. Although there were similarities in the order in which things were conducted at each institution, there was some variation in timing based on institutional context. |
| --- | --- |

| Phase 5: Defining and naming themes | Refining and naming these themes caused realization that they closely mirrored Kotter’s(3) principles for change. Without compelling reason to rename, we made decision to use Kotter’s steps and provide explication of how these steps are implemented in establishing ISS program. This action caused us to compare Kotter’s steps to our previous timeline, and we verified the fit. |
| --- | --- |

| Phase 6: Producing the report | Developed Table that contained Kotter’s step, Process applied to ISS, and Illustrative examples for each step. |
| --- | --- |

*ISS = In-situ simulation

1. Braun V, Clarke V. Using thematic analysis in psychology. Qualitative Research in Psychology. 2006;3(2):77-101.

2. Wendt M. Comparing ‘deep’ insider knowledge: developing analytical strategies for cross-national qualitative studies. International Journal of Social Research Methodology. 2020;23(3):241-54.

3. Kotter JP. Leading change: Why transformation efforts fail. Harvard Business Review. 2007;85(1):96-103.
